# Perinatal hypoxia: different effects of the inhibitors of GABA transporters GAT1 and GAT3 on the initial velocity of [^3^H]GABA uptake by cortical, hippocampal, and thalamic nerve terminals

**DOI:** 10.3325/cmj.2014.55.250

**Published:** 2014-06

**Authors:** Natalia Pozdnyakova, Marina Dudarenko, Ludmila Yatsenko, Nina Himmelreich, Olga Krupko, Tatiana Borisova

**Affiliations:** Department of Neurochemistry, Palladin Institute of Biochemistry, National Academy of Sciences of Ukraine, Kiev, Ukraine

## Abstract

**Aim:**

To analyze the effects of highly selective blocker GAT1, NO-711, and substrate inhibitor GAT3, β-alanine, on the initial velocity of [^3^H]GABA uptake by cortical, hippocampal, and thalamic nerve terminals (synaptosomes) after perinatal hypoxia.

**Methods:**

Animals were divided into two groups: control (n = 17) and hypoxia (n = 12). Rats in the hypoxia group underwent hypoxia and seizures (airtight chamber, 4% O_2_ and 96% N_2_) at the age of 10-12 postnatal days and were used in the experiments 8-9 weeks after hypoxia.

**Results:**

In cortical synaptosomes, the effects of NO-711 (30 μΜ) and β-alanine (100 μΜ) on [^3^H]GABA uptake were similar in control and hypoxia groups. In hippocampal synaptosomes, NO-711 inhibited 84.3% of the initial velocity of [^3^H]GABA uptake in normal conditions and 80.1% after hypoxia, whereas the effect of β-alanine was increased after hypoxia from 14.4% to 22.1%. In thalamic synaptosomes, the effect of NO-711 was decreased by 79.6% in controls and by 70.9% in hypoxia group, whereas the effect of β-alanine was increased after hypoxia from 20.2% to 30.2%.

**Conclusions:**

The effectiveness of β-alanine to influence GABA uptake was increased in hippocampal and thalamic nerve terminals as a result of perinatal hypoxia and the effectiveness of NO-711 in thalamic nerve terminals was decreased. These results may indicate changes in the ratio of active GAT1/GAT3 expressed in the plasma membrane of nerve terminals after perinatal hypoxia. We showed a possibility to modulate non-GAT1 GABA transporter activity in different brain regions by exogenous and endogenous β-alanine.

Perinatal hypoxia leads to multiple chronic neurological deficits including mental retardation, learning and memory disability, behavioral abnormalities, and even epilepsy (1). Pathological consequences of early life hypoxia may be a result of disturbance of the highly regulated maturation process. γ-Aminobutyric acid (GABA) as the first functional neurotransmitter in the developing brain fulfils an important signaling function in synapse formation and network construction (2). Hypoxic injury of the developing brain is mainly studied using the hypoxia model, in which rat pups undergo a brief exposure to graded global hypoxia in an airtight chamber (3). This showed a particular vulnerability of GABAergic neurons (4-6) and a long-lasting decrease in thresholds to convulsant action in adult rats that underwent hypoxia at an early age (3,6,7). In rats 10-12 postnatal days old, a single brief episode of moderately graded global hypoxia led to the development of tonic-clonic activity and caused a long-lasting (70-80 days after hypoxia) selective increase in seizure susceptibility in hippocampal slices (3).

Sodium- and chloride-dependent GABA transporters (GATs), which belong to the SLC6 superfamily of Na^+^ -dependent transporters, terminate inhibitory synaptic transmission, ie, after release from presynaptic nerve terminals GABA is rapidly removed from the extracellular space by GATs, thereby maintaining optimal ambient level of the neurotransmitter. Chronic neurological abnormalities, which develop after hypoxia at an early age, may be associated with changes in the functioning of GATs (8-10). Our previous experiments on rats that underwent perinatal hypoxia demonstrated a long-lasting increase in the ambient GABA level in cortical and hippocampal nerve terminals, whereas the thalamus was less sensitive to perinatal hypoxia, and thalamic GATs, in contrast to cortical and hippocampal ones, had a lower affinity to GABA (11).

Four types of GABA transporters are expressed in the plasma membrane of presynaptic nerve terminals and glial cells, that is, GAT1, GAT2, GAT3, and GAT4. GATs serve as one of the main targets for drugs in the treatment of neurological disorders, and so GABA uptake inhibitors are very promising agents with potential application in epilepsy, anxiety, pain, drug abuse, sleep disorders, and other disorders (8,9). The two most likely candidates for the maintenance of optimal ambient level of GABA in the brain are GAT1 and GAT3 (8). 1,2,5,6-Tetrahydro-1-(2-(((diphenylmethylene)amino)oxy)ethyl)-3-pyridinecarboxylic acid hydrochloride (NO-711) is a potent and selective GAT1 inhibitor with an IC_50_ of 0.38 µM; IC_50_ for GAT2 and GAT3 are 729 and 349 µM, respectively (12,13). Noteworthy, mixed GAT inhibitors are shown to have much broader spectrum of anticonvulsant activity than compounds with affinity only for GAT1. There are reports suggesting that non-GAT1 inhibitors are very interesting as potential candidates for future epilepsy treatment (8,14). Since the physiological role of GATs other than GAT1 is not fully determined, these inhibitors can also be pharmacological tools for the research on the biological role of non-GAT1 GABA transporters (15). In this context, there is a great need for the analysis of GABA uptake inhibitors, which can be used as research tools or potential drugs (16). β-alanine, a structural intermediate between α-amino acid (glycine, glutamate) and γ-amino acid (GABA) neurotransmitters, is a substrate inhibitor of GAT3. It should be underlined that exogenous β-alanine is carried across the blood brain barrier (BBB) into the central nervous system (CNS) via a taurine-sensitive β-amino acid transporter (17).

The aim of the research was to analyze the effects of highly-selective blocker of GAT1, NO-711, and substrate inhibitor of GAT3, β-alanine, on the initial velocity of [^3^H]GABA uptake by cortical, hippocampal, and thalamic nerve terminals isolated from control rats (8-9 weeks old) and rats (8-9 weeks old) that preliminary underwent hypoxia and seizures at the age of 10-12 postnatal days (perinatal hypoxia). The research question was how to modulate long-lasting changes in GABA uptake (and ambient level of GABA) in different brain regions after perinatal hypoxia/seizures taking into account that these changes might result from altered functioning of different types of GATs.

## Materials and methods

### Materials

β-Alanine, aminooxyacetic acid, N-2-hydroxyethylpiperazine-n-2-ethanesulfonic acid (HEPES), EDTA, D-glucose, sucrose, NO-711, Whatman GF/C filters, analytical grade salts were purchased from Sigma (St. Louis, MO, USA). [^3^H]GABA (94 Ci/mmol) and Organic Counting Scintillant (OCS) were received from Amersham (Little Chalfont, UK).

### Ethical considerations

Experiments were carried out in accordance with the European Guidelines and International Laws and Policies. We used Wistar rats (n = 29) from the vivarium of MD Strazhesko Institute of Cardiology, Medical Academy of Sciences of Ukraine. Animals were kept in the animal facilities of the Palladin Institute of Biochemistry of National Academy of Sciences of Ukraine, Kyiv, in accordance with the European Guidelines and International Laws and Policies (18). They were housed in a quiet, temperature-controlled room (22-23°C) and were provided with water and dry food pellets *ad libitum*. Rats were decapitated and the brain was removed. All procedures conformed to the guidelines of the Palladin Institute of Biochemistry. Before starting the experiments in 2012, the protocols were approved by the Animal Care and Use Committee of the Palladin Institute of Biochemistry (Protocol from 19/09-2012). All efforts were made to minimize animal suffering and use only the number of animals necessary to produce statistically significant data.

### Exposure to hypoxia

Wistar rats, males, were divided into two groups: vivarium control (n = 12) and hypoxia (n = 12), also 5 control animals were used for the analysis of time-dependence of inhibitory effects. At postnatal days 10-12, animals were removed from the litter and placed in an airtight chamber infused by atmosphere composed of 4% O_2_ and 96% N_2_. The exposure in the chamber lasted for 12 minutes until strongly pronounced tonico-clonic seizures developed (3). Animals exposed to hypoxia and their littermates were taken in experiments 8-9 weeks after the hypoxia episode.

### Isolation of synaptosomes

Control and experimental animals from each litter were analyzed simultaneously. After decapitation, the brain was quickly removed and immediately placed in ice-cold solution (0.32 M sucrose, 5 mM HEPES-NaOH, pH 7.4, 0.2 mM EDTA). Then the motor zone of the cortex, hippocampus, and thalamus were rapidly removed and homogenized in ice-cold solution (0.32 M sucrose, 5 mM HEPES-NaOH, pH 7.4, 0.2 mM EDTA) taken in the ratio of 1:10 (weight/volume). The homogenates were centrifuged (2500 g, 5 minutes) and the supernatants were carefully removed and again centrifuged at 15 000 g for 12 minutes for isolation of crude synaptosomal fraction. Purified synaptosomes were prepared by Ficoll-400 density gradient centrifugation of crude preparations according to the method of Cotman (19). Synaptosomes were suspended in the standard salt solution containing (in mM): NaCl, 126; KCl, 5; CaCl_2_, 1; MgCl_2_, 2; NaH_2_PO_4_, 1.0; HEPES-NaOH, 20, pH 7.4; D-glucose, 10, and used in the experiments during 2-4 hours after isolation. All buffers and synaptosomal suspensions were constantly oxygenated. All manipulations were performed at 0-4°C. Protein concentration was measured according to Larson et al (20) with bovine serum albumin as a standard.

### GABA uptake experiments

Synaptosomes were diluted with standard salt solution containing GABA transaminase inhibitor, aminooxiacetic acid 100 μM to minimize the formation of GABA metabolites. The concentration of synaptosomal protein in the samples was 100 μg/mL. Samples were preincubated for 15 minutes at 37°C and GABA/[^3^H]GABA (1 μM/50nM-0.1 μCi/mL, respectively) were added. GABA uptake was terminated in different time intervals by filtering aliquots through Whatman GF/C filters. After washing them twice with 5 mL ice-cold standard saline, filters were dried and suspended in Organic Counting Scintillant and counted in a Delta 300 scintillation counter (Tracor Analytic, Elk Grove Village, IL, USA). NO-711 and β-alanine were used as inhibitors of GABA uptake at a concentration of 30 μM and 100 μM, respectively. Non-specific binding of the neurotransmitter was evaluated in cooling samples sedimented immediately after the addition of radiolabelled GABA. Each measurement was performed in triplicate.

### Statistical analysis

Results are expressed as mean ± standard error of the mean of n independent experiments. Difference between two groups was compared by two-tailed *t*-test. Differences were considered significant at *Р*≤0.05.

## Results

### The effect of the incubation time on the inhibitory capacity of NO-711 and β-alanine

**Figure 1:**
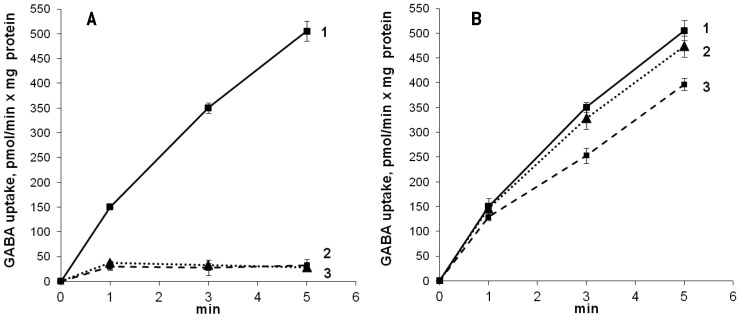
The effect of NO-711 (30 μM) (**A**) and β-alanine (100 μM) (**B**) on [^3^H]GABA uptake by cortical synaptosomes. [^3^H]GABA uptake: 1) control; 2) acute effects of the inhibitors, ie, NO-711 (**A**) or β-alanine (**B**) and [^3^H]GABA when they are simultaneously added to the medium; 3) preliminary incubation of synaptosomes with NO-711 (**A**) or β-alanine (**B**) for 15 minutes. Data are presented as mean ± standard error of the mean of three independent experiments, each performed with different synaptosomal preparations in triplicate.

In the first sets of the experiments, we assessed how the incubation time of synaptosomes with the inhibitors influenced [^3^H]GABA uptake. Using cortical synaptosomes, it was shown that acute effect of NO-711 (that is, simultaneous addition of NO-711 (30 μΜ) and [^3^H]GABA, which starts the uptake process) and the effect after preliminary incubation with synaptosomes for 15 minutes were similar. NO-711 decreased synaptosomal accumulation of [^3^H]GABA for 5 minutes by 94.3% in acute treatment and 93.6% after 15-minute preincubation (Figure 1A).

The effect of β-alanine, used at a concentration of 100 μΜ, was also studied during acute treatment and under conditions of preliminary incubation with synaptosomes. Figure 1, B shows the time course of [^3^H]GABA uptake by cortical synaptosomes in the presence of β-alanine. The inhibitor added to synaptosomes simultaneously with [^3^H]GABA slightly decreased the initial velocity of [^3^H]GABA uptake (by 3.0%) and [^3^H]GABA accumulation for 5 minutes (by 6.3%) (Figure 1B, curve 2). However, the preliminary incubation of synaptosomes with β-alanine for 15 minutes decreased the initial velocity of uptake and accumulation of [^3^H]GABA for 5 minutes by 14.1% and 21.4%, respectively, thereby increasing its inhibitory effect (Figure 1B, curve 3). Similar dependence of inhibitory capacity of NO-711 and β-alanine on the incubation time was observed in the experiments with hippocampal and thalamic synaptosomes (data not shown).

In the next series of the experiments, the effect of NO-711 and β-alanine on the initial velocity of [^3^H]GABA uptake by cortical, hippocampal, and thalamic synaptosomes isolated from control rats and rats that underwent hypoxia was analyzed. The protocol included preliminary incubation of synaptosomes with 30 μΜ NO-711 and 100 μΜ β-alanine for 15 minutes.

### The effect of NO-711 on [^3^H]GABA uptake by cortical, hippocampal, and thalamic synaptosomes of control rats and rats exposed to perinatal hypoxia

**Figure 2:**
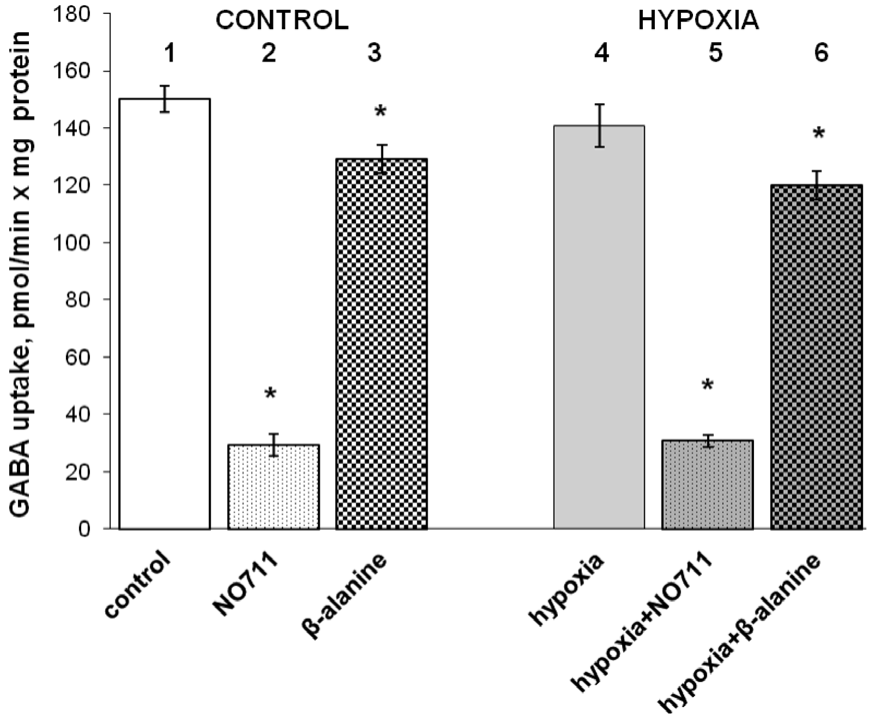
The effects of NO-711 at a concentration of 30 μΜ (15 minutes preincubation) (the second and fifth column) and β-alanine at a concentration of 100 μΜ (15 minutes preincubation) (the third and sixth column) on the initial velocity of [^3^H]GABA uptake by cortical synaptosomes isolated from control rats (the first triplet of columns) and rats preliminarily exposed to hypoxia and seizures at the age of 10-12 postnatal days (the second triplet of columns). Data are presented as mean ± standard error of the mean of three independent experiments, each performed with different synaptosomal preparations in triplicate. Asterisk – *Р*≤0.05 as compared to control.

*Cortical nerve terminals.* In the control group, the initial velocity of [^3^H]GABA uptake was decreased by 80.1%, from 150.2 ± 2.4 pmol/min x mg of protein to 29.3 ± 3.9 pmol/min x mg of protein in the presence of NO-711 (Figure 2, the first and second column).

In rats exposed to hypoxia, the initial velocity of [^3^H]GABA was decreased by 9% in comparison with the normal level (Figure 2, the first and fourth column). The initial velocity of [^3^H]GABA uptake was 140.8 ± 1.9 pmol/min x mg of protein without NO-711 and 30.7 ± 2.1 pmol/min x mg of protein with NO-711 (78.2% decrease). Therefore, the effect of NO-711 on [^3^H]GABA uptake was similar in control animals and animals exposed to hypoxia (Figure 2, the second and fifth column).

**Figure 3:**
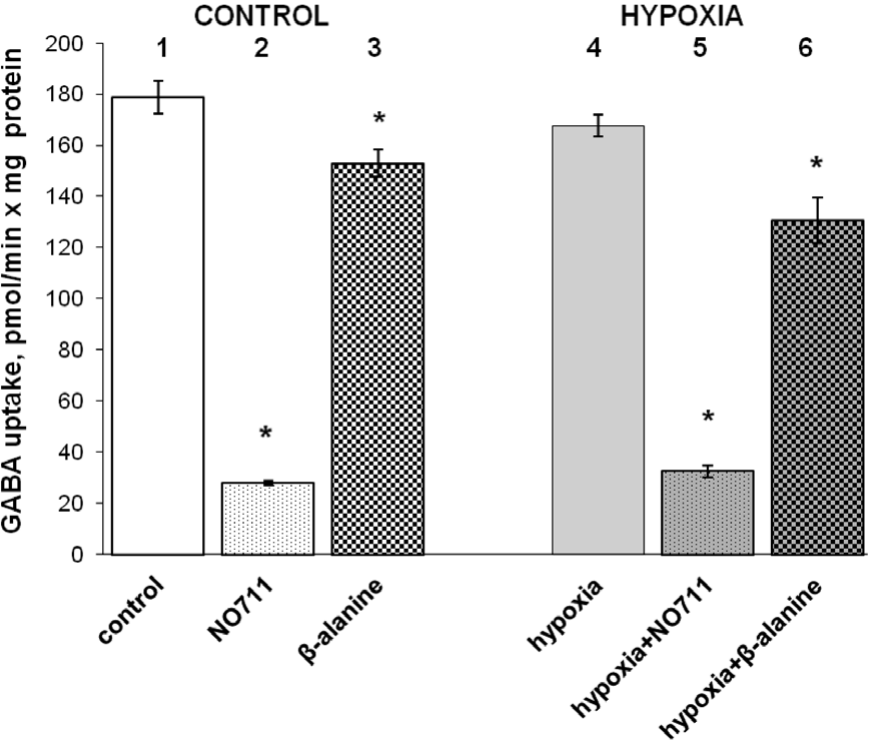
The effects of NO-711 at a concentration of 30 μΜ (15 minutes preincubation) (the second and fifth column) and β-alanine at a concentration of 100 μΜ (15 minutes preincubation) (the third and sixth column) on the initial velocity of [^3^H]GABA uptake by hippocampal synaptosomes isolated from control rats (the first triplet of columns) and rats preliminarily exposed to hypoxia and seizures at the age of 10-12 postnatal days (the second triplet of columns). Data are presented as mean ± standard error of the mean of three independent experiments, each performed with different synaptosomal preparations in triplicate. Asterisk – *Р*≤0.05 as compared to control.

*Hippocampal nerve terminals.* In the control group, the initial velocity of [^3^H]GABA uptake was decreased by 84.3%, from 178.6 ± 2.8 pmol/min x mg of protein to 28.0 ± 1.1 pmol/min x mg of protein in the presence of NO-711 (Figure 3, the first and second column).

In rats exposed to hypoxia, the initial velocity of [^3^H]GABA uptake was by 6% lower than the normal level (Figure 3, the first and fourth column) and was 167.7 ± 2.1 pmol/min x mg of protein. In the presence of NO-711, it was 32.6 ± 1.2 pmol/min x mg of protein (Figure 3, the fifth column). So, NO-711 decreased it by 80.1%. Therefore, the effectiveness of NO-711 to influence [^3^H]GABA uptake decreased in animals exposed to hypoxia in comparison with the control animals (Figure 3, the second and fifth column).

**Figure 4:**
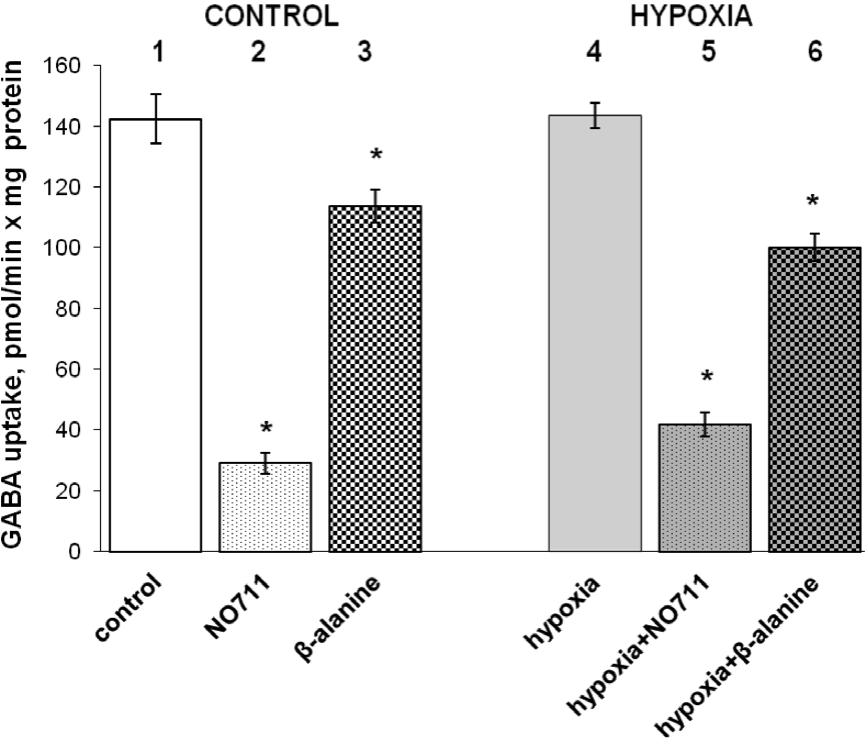
The effects of NO-711 at a concentration of 30 μΜ (15 minutes preincubation) (the second and fifth column) and β-alanine at a concentration of 100 μΜ (15 minutes preincubation) (the third and sixth column) on the initial velocity of [^3^H]GABA uptake by thalamic synaptosomes isolated from control rats (the first triplet of columns) and rats preliminary exposed to hypoxia and seizures at the age of 10-12 postnatal days (the second triplet of columns). Data are presented as mean ± standard error of the mean of three independent experiments, each performed with different synaptosomal preparations in triplicate. Asterisk – *Р*≤0.05 as compared to control.

*Thalamic nerve terminals* In the control group, NO-711 decreased the initial velocity of [^3^H]GABA uptake by 79.6%, from 142.4 ± 4.8 pmol/min x mg of protein to 29.0 ± 2.1 pmol/min x mg of protein in (Figure 4, the first and second column).

Exposure to hypoxic conditions did not significantly change the initial velocity of [^3^H]GABA uptake by thalamic synaptosomes in comparison with the normal level (Figure 4, the first and fourth column), which was 143.5 ± 4.2 pmol/min x mg of protein. In the presence of NO-711, it was 41.8 ± 3.4 pmol/min x mg of protein (Figure 4, the fifth column) (70.9% reduction). Therefore, the effectiveness of NO-711 was decreased in animals exposed to hypoxia in comparison with the control animals (Figure 4, the second and fifth column).

### The effect of β-alanine on [^3^H]GABA uptake by cortical, hippocampal, and thalamic synaptosomes of control rats and rats exposed to hypoxia

*Cortical nerve terminals.* In the control group, the initial velocity of [^3^H]GABA uptake in the presence of β-alanine was decreased by 14.1% and was 129.1 ± 3.8 pmol/min x mg of protein (Figure 2, the first and third column).

After exposure to hypoxic conditions, the initial velocity of [^3^H]GABA uptake was 120.0 ± 3.1 pmol/min x mg of protein (Figure 4, the sixth column) and β-alanine decreased it by 15.1%. Therefore, the effectiveness of β-alanine was not changed significantly in animals exposed to hypoxia compared with control animals (Figure 2, the third and sixth column).

*Hippocampal nerve terminals.* In the control group, the initial velocity of [^3^H]GABA uptake in the presence of β-alanine was decreased by 14.4% and was 152.9 ± 4.3 pmol/min x mg of protein (Figure 3, the first and third column).

Under hypoxic conditions, the initial velocity of [^3^H]GABA uptake in the presence of β-alanine was 130.7 ± 3.2 pmol/min x mg of protein (Figure 3, the sixth column). β-alanine decreased the initial velocity of [^3^H]GABA uptake by 22.1%. Therefore, the effectiveness of β-alanine was increased in animals exposed to hypoxia in comparison with the control animals (Figure 3, the third and sixth column).

*Thalamic nerve terminals.* In the control group, the initial velocity of [^3^H]GABA uptake in the presence of β-alanine was lower by 20.2% and was equal to 113.6 ± 3.3 pmol/min x mg of protein in the presence of β-alanine (Figure 4, the first and third column).

After hypoxia, β-alanine decreased the initial velocity of [^3^H]GABA uptake by 30.2% to 100.1 ± 2.5 pmol/min x mg of protein (Figure 4, the sixth column). Therefore, the effectiveness of β-alanine was increased in animals exposed to hypoxia in comparison with the control animals (Figure 4, compare the third and sixth column).

## Discussion

GABA plays a key role in the regulation of neuronal excitability maintaining the inhibitory tone that counterbalances neuronal excitation. A disturbance in GABAergic signaling is involved in the pathogenesis of several CNS diseases, including anxiety, sleep disorders, epilepsy, depression, chronic pain, and others. A promising approach in the successful treatment of these disorders is the use of drugs influencing GABA uptake. Compounds addressing GAT1 and GAT3 are of special interest for enhancing GABA neurotransmission, and this is especially true for potent and selective GAT3 inhibitors as these are lacking so far (21). Mixed GAT inhibitors have much broader spectrum of anticonvulsant activity than compounds with affinity only for GAT1 (8,14). Until now, only tiagabine – a selective inhibitor of GAT1 transporter has been used in the clinic in the treatment of partial seizures. It has been also shown to be effective in a variety of non-epileptic conditions, including psychosis, general anxiety and sleep disorders, bruxism, drug addiction, acute and chronic pain, tonic spasm, posttraumatic stress, essential tremor, and migraine prophylaxis (16).

Hypoxia and seizures early in life can cause multiple neurological deficits and even chronic epilepsy. Imbalance between excitation and inhibition is considered to be a crucial factor in the etiology of neurological disorders arising after hypoxia and seizures at an early age. So, it is extremely important to keep appropriate extracellular concentration of excitatory and inhibitory neurotransmitters between episodes of regulated exocytosis, ie, fusion of synaptic vesicles containing neurotransmitter with the plasma membrane (11,22-26). It is still unclear how to modulate long-lasting changes in GABA uptake and ambient GABA level in different brain regions after perinatal hypoxia/seizures if these changes result from altered functioning of different types of GATs.

The initial velocity of high-affinity [^3^H]GABA uptake and accumulation of [^3^H]GABA were assessed in isolated rat brain nerve terminals (synaptosomes), which retain all features of intact nerve terminals, eg, ability to maintain the membrane potential and exocytotic release, as well as accomplish the uptake of the neurotransmitters. Synaptosomes promise to be one of the best systems to explore the relationship between the structure of a protein, its biochemical and cell-biological properties, and physiological role (27). We demonstrated a decrease in the initial velocity of [^3^H]GABA uptake in cortical and hippocampal nerve terminals isolated from rats 8-9 weeks old that preliminary underwent hypoxia and seizures at the age of 10-12 postnatal days in comparison with control rats, but we found no alterations in the thalamus. These data are in accordance with our previous results on the assessment of the extracellular level of [^3^H]GABA in nerve terminals after perinatal hypoxia, where we found a long-lasting increase in the ambient [^3^H]GABA level in cortical and hippocampal nerve terminals, whereas the thalamus was less sensitive (11). Taking into account that the main types of GABA transporters, which determine the optimal ambient level of GABA in the brain, are GAT1 and GAT3, we analyzed the effects of highly selective blocker of GAT1, NO-711, and substrate inhibitor of GAT3, β-alanine, on [^3^H]GABA uptake by nerve terminals. It was shown that the inhibitory capacity of NO-711 and β-alanine was not changed after perinatal hypoxia in cortical synaptosomes. The inhibitory capacity of β-alanine to influence GABA uptake was increased in hippocampal and thalamic nerve terminals as a result of perinatal hypoxia, whereas the capacity of NO-711 in thalamic nerve terminals was decreased. Taking into account our data on the effect of NO-711 and β-alanine in the thalamus, it may be speculated that surface expression of GAT1 and GAT3 was changed after perinatal hypoxia, thereby representing the compensatory mechanism underlying the maintenance of the optimal ambient level of GABA. In contrast, there were no alterations in the inhibitory capacity of NO-711 and β-alanine in the cortex that may reflect the unchanged ratio GAT1/GAT3 after perinatal hypoxia. Recently, we have shown that perinatal hypoxia did not change the affinity of GATs to substrate in the cortex, hippocampus, and thalamus (11), so it may be suggested that alteration in the effectiveness of the inhibitors after perinatal hypoxia is more associated with the changes in the number of the transporters in the plasma membrane than with the affinity of the inhibitors to substrate-binding site of GATs.

It is not new that hypoxia changes the expression of different types of GATs. Melone et al (28) showed that transient focal ischemia triggered a novel neuronal expression of GAT3 in the rat perilesional cortex. Dalby et al (8) suggested that the ratio of GAT1/GAT3 affinity was very important for the efficacy of non-selective GABA uptake inhibitors in epilepsy. GAT3-mediated GABA uptake comes into play only under specific physiological or pathological conditions, which results in an increase in neuronal activity and GABA release beyond normal levels in the striatum (29). It was demonstrated that protein kinase C activation induced serine/threonine phosphorilation of GAT1, which in turn promoted GAT1 internalization, and also in the presence of tyrosine kinase activators the insertion of GAT1 to the plasma membranes was accelerated (30).

Experiments with exogenously applied β-alanine are especially interesting because this molecule can be readily absorbed from the gastrointestinal tract and transported into the CNS. β-alanine is carried across the BBB into the CNS via a taurine-sensitive β-amino acid transporter in a Na^+^ and Cl^-^-dependent manner. This transporter is highly selective for β-amino acids because neither L-glutamate (transported by the anionic amino acid transporter) nor L-phenylalanine (transported by the large neutral amino acid transporter) affects the movement of β-alanine across the BBB. β-alanine may also cross the BBB by passive diffusion (17). Analogues of β-alanine are proposed for a treatment of epilepsy and as a basis for antiepileptic drug design (31). When orally administered, plasma concentrations of β-alanine rise rapidly, peaking within 30-45 minutes, then dropping significantly within 90-120 minutes (32). Despite being a simple amino acid, β-alanine remains essentially unexplored as either a neurological drug or a drug design platform (33).

Our data on the effects of β-alanine can be interesting also because β-alanine exhibits physiological significance. β-alanine is widely distributed throughout the brain, and is one of only a few naturally occurring β-amino acids endogenous to humans and mammals. The average concentrations of β-alanine within the brain are between 0.03 and 0.08 mM. A detailed examination of β-alanine concentrations in rat brain has shown that β-alanine shows regional differences: midbrain contains the highest concentration (0.108 mmol/g frozen weight); the cortex contains just over half of this value (0.065 mmol/g), while the cerebellum contains the lowest concentration (0.039 mmol/g). Regional distribution of β-alanine in the CNS is an important attribute of a neurotransmitter substance (17). This variation of β-alanine concentrations in the CNS is similar to variable concentrations for GABA (12). In the CNS, β-alanine acts as a depressant of neuronal activity with the potency comparable to that of the neurotransmitter GABA. Nevertheless, the role of β-alanine in sleep, consciousness, epilepsy, stroke, and other disorders requires greater clarification (17). In this context, our data may be also of value for the analysis of possible inter-synaptic crosstalk between GABA- and β-alanine-ergic nerve terminals. In this case, released β-alanine can cause inhibition of non-GAT1-mediated uptake of GABA. Moreover, Mathers et al (34) has recently shown that chemical transmission at the inhibitory synapses in the thalamus involves receptor activation by β-alanine. The importance of β-alanine for synaptic transmission is supported by the fact that the patients with β-alaninemia (accumulation of β-alanine as inborn error of metabolism) may develop neurological abnormalities whose mechanisms are far from being understood (35).

It has to be underlined that except GABA transporters, β-alanine has five recognized receptor sites: glycine coagonist site on the NMDA (N-methyl-D-aspartate) complex; glycine receptor site; GABA-A receptor, and GABA-C receptor (33). As imbalance between excitation and inhibition is considered a crucial factor in the etiology of neurological disorders arising after hypoxia and seizures at early age, there may be a wide range of β-alanine applications including complementary regulation of GABA- and glutamate-ergic neurotransmission.

Therefore, we showed that the efficacy of β-alanine to inhibit GABA uptake was increased in hippocampal and thalamic nerve terminals as a result of perinatal hypoxia, whereas the capacity of NO-711 in thalamic nerve terminals was decreased. This might result from the alterations in the ratio of active GAT1/GAT3 expressed in the plasma membrane of nerve terminals after perinatal hypoxia. This fact shows a possibility of targeted modulation of GAT3 transporter activity and GABAergic neurotransmission under pathological conditions by exogenous and endogenous naturally occurring β-alanine.
